# Supplementary material for: A 3D navigation template for guiding a unilateral lumbar pedicle screw with contralateral translaminar facet screw fixation: a study protocol for multicentre randomised controlled trials
Source: BMJ Open. 2017 Jul 21;7(7):e016328. doi: 10.1136/bmjopen-2017-016328 (PMC5642762; doi:10.1136/bmjopen-2017-016328)
Supplement: Author's manuscript [file bmjopen-2017-016328.draft_revisions.pdf]

BMJ Open

The 3D navigation template in guiding unilateral lumbar pedicle screw with contralateral translaminar facet screw fixation: A study protocol of multicenter randomized controlled trials.

Journal:	*BMJ Open*
Manuscript ID	bmjopen-2017-016328
Article Type:	Protocol
Date Submitted by the Author:	10-Feb-2017
Complete List of Authors:	Shao, Zhen-Xuan; Orthopaedic Hospital, The Second Affiliated Hospital and Yuying Children's Hospital of the Wenzhou Medical University, The Second Medical School of the Wenzhou Medical University, Zhejiang Spine Center, Wenzhou, China., Department of Spine Surgery<br>He, Wei; People´s Hospital of Shaoxing, Zhejiang University Shaoxing Hospital, Shaoxing, China., Department of Orthopaedics<br>He, Shao-Qi; People´s Hospital of Shaoxing, Zhejiang University Shaoxing Hospital, Shaoxing, Zhejiang, China., Department of Orthopaedics<br>Lin, Sheng-Lei; Wenzhou Center Hospital, Dingli Hospital of Wenzhou Medical University, Wenzhou, China., Department of Orthopaedics<br>Huang, Zhe-Yu; Ningbo No. 6 Hospital, Ningbo, Zhejiang, China, Department of Orthopaedics<br>Tang, Hong-Chao; Jiaxing Hospital of Zhejiang General Corps of Armed Police Forces, Jiaxing, China, Department of Orthopaedics<br>ni, wenfei<br>Wang, Xiang-Yang; Second Affiliated Hospital of Wenzhou Medical University, Second Medical College of Wenzhou Medical University<br>Wu, Ai-Min; Orthopedics
**Primary Subject Heading**:	Surgery
Secondary Subject Heading:	Evidence based practice
Keywords:	3D navigation template, Translaminar facet screw fixation, Multicenter randomized controlled trials, Study protocol

SCHOLARONE™ Manuscripts

Title: The 3D navigation template in guiding unilateral lumbar pedicle screw with contralateral translaminar facet screw fixation: A study protocol of multicenter randomized controlled trials.

Authors: Zhen-Xuan Shao[1] MD; Wei-He[2] MD; Shao-Qi He[3] MD; Sheng-Lei Lin[4] MD; Zhe-Yu Huang[5] MD; Hong-Chao Tang MD[6], Wen-Fei Ni[1] MD PhD; Xiang-Yang Wang[1] MD PhD; Ai-Min Wu[1]* MD PhD;

Affiliated:

1. Department of Spine Surgery, Orthopaedic Hospital, The Second Affiliated Hospital and Yuying Children's Hospital of the Wenzhou Medical University, The Second Medical School of the Wenzhou Medical University, Zhejiang Spine Center, Wenzhou, China.

2. Department of Orthopaedics, People´s Hospital of Shaoxing, Zhejiang University Shaoxing Hospital, Shaoxing, China.

3. Department of Orthopaedics, People's Hospital of Ruian, The Third Affiliated Hospital of Wenzhou Medical University, Ruian, China.

4. Department of Orthopaedics, Wenzhou Center Hospital, Dingli Hospital of Wenzhou Medical University, Wenzhou, China.

5. Department of Orthopaedics, Ningbo No. 6 Hospital, Ningbo, China.

6. Department of Orthopaedics, Jiaxing Hospital of Zhejiang General Corps of Armed Police Forces, Jiaxing, China.

For peer review only

Funds and Acknowledgement: This work was funded by the National Natural Science Foundation of China (81501933), Zhejiang science and technology innovation project (2016R413061), Wenzhou Science and Technology Project (Y20160369). The funders had no role in the design, execution, or writing of the study.

Disclosures: All authours declare that they have no conflict of interest.

Author contributors: XYW and AMW is the principal investigator of this trial. ZXS, WFN, XYW, and AMW contributed to the development of the research question and trial design. ZXS and AMW contributed to the drafting of the manuscript, ZXS, WH, SQH, SLL, ZYH and HCT are responsible for the recruitment and treatment of patients. All authors edited the final manuscript. All authors read and approved the final manuscript.

Data sharing statement: The full data set will be made available when this trial is completed and published. Application for the data to be released should be made in contact to AMW (principle investigator).

***Corresponding author:**

Ai-Min Wu MD PhD E-mail: aiminwu2005@163.com

Department of Spine Surgery, Orthopaedic Hospital, The Second Affiliated Hospital and Yuying Children's Hospital of the Wenzhou Medical University, The Second Medical School of the Wenzhou Medical University, Zhejiang Spine Center, Wenzhou, China. **Address:** 109# Xueyuan Xi Road, Wenzhou, China, 325027. **Phone:** 8613758745616, **Fax:** 8657788002823.

For peer review only

The 3D navigation template in guiding unilateral lumbar pedicle screw with contralateral translaminar facet screw fixation: A study protocol of multicenter randomized controlled trials.

Abstract

Introduction: The incidence of lumbar disc degeneration disease is increasing in recent years. The lumbar interbody fusion using unilaterally two pedicle screws and a translaminar facet screw fixation have advantages of minimally invasive and less cost when compared to the traditional methods. Meanwhile, the method guided by the 3D navigation template may help us improve the accuracy and the success rate of surgery. This is the first randomized study using the 3D navigation template in guiding unilateral lumbar pedicle screw with contralateral translaminar facet screw fixation.

Methods and Analysis: Patients who meet the criteria of the surgery will be randomly divided into experimental group and control groups by computer-generated randomisation schedule. We will design an individual 3D navigation template by CATIA software and MeditoolCreate preoperatively. The primary outcomes will be collected: screw angles compare to the optimal screw trajectories in 3D digital images; length of wound incision; operative time; intra-operative blood loss and complications. The secondary outcomes will be collected: Visual Analog Scale of back pain (VAS of back pain); Visual Analog Scale of leg pain (VAS of leg pain); Oswestry Disability Index (ODI), these parameters will be evaluated on day 1, then 3, 6, 12 and 24 months postoperatively.

For peer review only - http://bmjopen.bmj.com/site/about/guidelines.xhtml

Ethics and dissemination: The study had been reviewed and approved by the IRB of Second Affiliated Hospital and Yuying Children's Hospital of the Wenzhou Medical University. The results will be presented in peer-reviewed journals and international spine related meeting after the study completed.

Trial registration number: ChiCTR-IDR-17010466

Keywords: 3D navigation template; Translaminar facet screw fixation; Multicenter randomized controlled trials; Study protocol.

Strengths and limitations of this study

1. This is the first multicenter randomized controlled trials to compare 3D navigation template in guiding unilateral lumbar pedicle screw with contralateral translaminar facet screw fixation vs. traditional multiple X-rays fluoroscopy.

2. We firstly using Mimics and CATIA software to design the 3D navigation template of unilateral lumbar pedicle screw with contralateral translaminar facet screw fixation.

3. The clinical data will be collected prospectively at least 24 months after operation.

4. Because this is the multicenter randomized controlled trials, the surgeons experiment in different research sites may be influence the outcomes.

For peer review only - http://bmjopen.bmj.com/site/about/guidelines.xhtml

Introduction

With the aging society, the incidence of lumbar disc degeneration disease is increasing [1 2], and which one of the major socioeconomic burden [3-5]. If conservative treatment of lumbar degenerative disease is ineffective, surgical intervention is recommended for these patients [6-9]. Compared to the pedicle screw fixation on both sides which gains popularity among general surgeons because of easiness, the lumbar interbody fusion using unilaterally two pedicle screws and a translaminar facet screw fixation can reduce tissue trauma, length of wound incision and promote recovery[10-12].

The translaminar screw is that a long screw goes through the spinous process, zygapophyseal joints and ends in lower vertebral facet to fix the other side of the joint[13]. Biomechanical properties of the translaminar screw are the same as pedicle screws through vitro biomechanical experiments[14 15]. Park et al[16] reported VAS and ODI scores of the patient were significantly improved, after minimally invasive surgery of anterior lumbar interbody fusion combined with the translaminar screw. Liu et al [11] reported recovery rate was 93.5% through minimally invasive surgery of transforaminal lumbar interbody fusion combined with the translaminar screw.

However, there is higher risk of nerve and vascular injury when the angle deviation of the translaminar screw or screw entry point is incorrect[17]. In order to achieve an optimal screw trajectory, many surgeons choose multiple X-rays images guide to locate the entrance point and adjust the angle, which will expose both patients and surgeons in considerable radiation[11 12 18]. The technique of 3D reconstruction and 3D rapid printed navigation template, which extracted the posterior surface futures of

For peer review only - http://bmjopen.bmj.com/site/about/guidelines.xhtml

lumbar vertebra, may help us make the optimal trajectory of translaminar facet screw fixation.

To the best of our knowledge, there was still none of randomized controlled studies of 3D navigation template vs. traditional multiple X-rays fluoroscopy to guide unilateral lumbar pedicle screw with contralateral translaminar facet screw fixation. In this study, we will conduct a randomized controlled trial (RCT) to compare 3D navigation template in guiding unilateral lumbar pedicle screw with contralateral translaminar facet screw fixation vs. traditional multiple X-rays fluoroscopy.

Methods and Analysis

The study had been reviewed and approved by the Institutional Ethics Review Board of Second Affiliated Hospital and Yuying Children's Hospital of the Wenzhou Medical University. All of participants will be sign the informed consent. The protocol has been registered in Chinese Clinical Trial Registry (ChiCTR), asigned to be the representative registry of China to join WHO ICTRP in 2007, with the protocol number: ChiCTR-IDR-17010466. The SPIRIT checklist 2013 (**Supplementary Table S1**) was used and check our reports[19 20].

Participants

This study is a parallel group RCT conducted at the Orthopaedic Hospital, Second Affiliated Hospital and Yuying Children's Hospital of the Wenzhou Medical University.

For peer review only - http://bmjopen.bmj.com/site/about/guidelines.xhtml

Inclusion criteria:

1. Adult patients with age more 18 years old;

2. Chronic low back pain or combined with neurological symptoms of lower extremities;

3. Single-segment lower lumbar vertebral disease (Including lumbar spinal cannal stenosis, foraminal stenosis, segmental instability, lumbar disc herniation, and painful disc degeneration (back disc));

4. Inefficacy after strict conservative treatment for more than 6 months.

Exclusion criteria:

1. Serious deformity of lumbar vertebrae;

2. Dysplasia of lumbar pedicle or vertebral laminar;

3. Obvious osteoporosis of lumbar vertebrae;

4. Metabolic bone diseases such as osteomalacia or Paget's disease;

5. Spondylolisthesis>grade 2 (Meyerding);

6. Caudaequinainjury or severe radiculopathy;

7. Post inflammatory instability of the vertebral spine;

8. Body mass index >30;

9. Immunologic diseases or Metabolic syndrome;

10. Therapy with systematic corticosteroids or immunosuppressants;

11. Current using Coumadin (or Warfarin) or Heparin therapy for more than 6 months at the time of operation.

For peer review only - http://bmjopen.bmj.com/site/about/guidelines.xhtml

Sample size calculation

We performed a power analysis in order to assess the required sample size to show safety with a power (1-b) of 0.8 and an α of 0.05. Under the pre experiment and related literature[21], the proportion of the control group was set to 84.6% and the proportion of intervention group was set to 95%. We made Two Independent Proportions Power Analysis on PASS (Power Analysis and Sample Size) and the result was 132.

Randomization and blinding

Patients will be randomly divided into experimental group and control group by computer-generated randomization schedule. And the random sequence will be stored in a sealed opaque envelope to ensure that patients, personnel and the outcome assessor will be blinded to group allocation. Until the last questionnaires have been completed, the participant's allocated intervention will be revealed by the study secretary. If a patient's condition deteriorates, stop the blinding experiment and attach heavy important to the patient's safety (**Figure 1**).

Monitoring

After screening, patients met the inclusion criteria should be participated in the study as far as possible. If not, we have to save the record for judgment of bias. Meanwhile, recording the patient's basic information is necessary to discuss the result

For peer review only - http://bmjopen.bmj.com/site/about/guidelines.xhtml

of the experiment.

Before the experiment, explain the purpose of the experiment, and the importance of the experimental procedures to obtain consent and ideal compliance. If the selected patient is unqualified, elimination is undesirable and the data of the patient is substituted by the average of the group. And loss to follow-up is inevitable; the data of these patients are also replaced by average.

Surgeons and data collectors will undergo training and preliminary experiment of data measurement and questionnaire will be carried out in advance. Recording the time and staff is necessary when collector collect and gather the data, which is convenient to review.

Study procedure

A retrospective review will be done on all patients by examining their medical records before operation. The involved segments were identified via a C-arm machine and then marked on the skin of the patient. The patients' CT images with a thickness less than 1 mm will be exported from the Star PACS image system of the hospital and imported into the Mimics 16.0 to constructing 3D images, in which we adjust the threshold depending on bone density. And we regard the two adjacent segments as a functional segmental unit (FSU) , such as L2-3, L3-4, L4-5, L5-S1.

Then, the individual 3D navigation templates will be designed and printed by CATIA software and MeditoolCreate. The detailed steps are as follows: The FSUs digital data in the Mimics 16.0 will be saved in STL format and imported into CATIA

For peer review only - http://bmjopen.bmj.com/site/about/guidelines.xhtml

software. We will establish the optimal screw trajectory, which include two vertebral pedicle screws trajectories and one contralateral translaminar facet screw trajectory. We will measure three angles of translaminar facet screw trajectory: α, β, γ. α is the angle between the screw trajectory and the vertical line in the anteroposterior view; β is the angle between the screw trajectory and the upper endplate of lower vertebra in the lateral view; γ is the angle between the screw trajectory and midline of the lumbar vertebra in the top view (**Figure 2**). The pedicle screws trajectories will be designed according to method of Roy-Camille[12]. We will measure two angles δ and ε, which is the angle between the ideal screw trajectory and midline of the lumbar vertebra in the top view, respectively (**Figure 2**).

After the optimal screw trajectories were determined, the curved posterior surface of the lumbar vertebra around the screw entry point was extracted and to create the 3D navigation template (**Figure 3**). Then we import the 3D navigation template file into the 3D printer to rapid print it through MeditoolCreate [13] (**Figure 4**).

The 3D navigation template will be sterilized and used to guide the screw in further operation on patients. In experimental group: the dissection of the paraspinal tissue will performed at one side to expose the part of spinous process, laminar, and facet joint, 3D navigation template will be attach the surface of above bony construction. Then the kirschner wire (K-wire) will be inserted inside of the 3D navigation template, and a trajectory for the screw will be fashioned with a cannulated drill, screws will be introduced after then. In control group: The multiple X-rays fluoroscopy will be used, and the screw trajectory will be adjusted by intra-operative X-rays fluoroscopy.

For peer review only - http://bmjopen.bmj.com/site/about/guidelines.xhtml

After operation, the CT scans of the patients in both groups will be obtained, the angels of α1, β1, γ1 δ1 and ε1 will be measured corresponding to the angles of the optimal screw trajectory in 3D images. The placement and position of the screws will be divided into three types (type I, the screw not penetrate out the cortex; type II, the screw partially penetrate out the cortex, but less than 2 mm; type III, the screw penetrate out the cortex and more than 2 mm).

Outcome measure

The primary outcomes:

1. Screw angles compare to the optimal screw trajectories in 3D digital images, and the position of the screws

2. Wound incision length, operative time, intra-operative blood loss, times of X-ray exposure and complications.

The secondary outcomes:

1. Visual Analog Scale of back pain (VAS of back pain)

2. Visual Analog Scale of leg pain (VAS of back pain)

3. Oswestry Disability Index (ODI)

And the data will be collected at preoperatively, day 1 postoperatively, then 3, 6, 12 and 24 months postoperatively.

Statistical analysis

All data will be analyzed using SPSS 19.0 software. The differences of wound

For peer review only - http://bmjopen.bmj.com/site/about/guidelines.xhtml

incision length, operative time, intra-operative blood loss, angles between two groups will be analyzed by two independent-samples t-test with α of 0.05. VASs and ODI scores at pre-operation, post-operation, 3 months post-operatively, 6 months postoperatively and 12 months postoperatively will be analyzed by the repeated-measures analysis of variance (ANOVA). The times of X-ray exposure and frequency of screw penetrate out the cortex will be compared by Chi square test.

Discussion

Translaminar facet screw fixation was reported as a minimally invasive technique[11 12 22], the Biomechanical comparisons between facet screw and pedicle screw fixation have been reported the equivalence biomechanically between them[14 15 23]. Current computer-assisted surgery systems may can achieve the accurate screw trajectory, however, the cost of computer-assisted surgery systems is considerable, and the manipulate is too complicated[24], whereas the accuracy remains dependent on multiple radiation.

Combining 3D reconstruction and 3D rapid printing is a new mode of production, which is applied in many fields because of the advantages of visualization, plasticity and rapid printing [25-27]. 3D printing templates can also help the surgeons to achieve an accurate screw placement of posterior cervical fixation and scoliosis [28 29].

In this study, we will compare the 3D navigation template to multiple X-rays fluoroscopy in guiding unilateral lumbar pedicle screw with contralateral translaminar facet screw fixation. The main strength of the study is the design as a RCT rather than

For peer review only - http://bmjopen.bmj.com/site/about/guidelines.xhtml

an observational comparative study. A RCT has the advantage to control all possible variables due to the random sequence generation as opposed to observational studies where confounding and bias may be more problematic. High-quality RCTs are generally regarded as the gold standard for studying the effectiveness of an intervention.

Because of some patients' death, relocation or other reasons, the representative of the sample is destroyed. Meanwhile, if some patients accept the experimental treatment measures such as therapeutic drugs, the results will appear deviation. As for these experiment biases, strict monitoring can't only reduce the loss of follow-up, but also improve patient compliance, by which bias will decrease.

Ethics and dissemination:

The study had been reviewed and approved by the Institutional Ethics Review Board of the Second Affiliated Hospital and Yuying Children's Hospital of the Wenzhou Medical University. The procedure will be performed following the principles described in the Declaration of Helsinki. All of participants will be signed the informed consent. The protocol has been registered in Chinese Clinical Trial Registry (ChiCTR), assigned to be the representative registry of China to join WHO ICTRP in 2007, with the protocol number ChiCTR-IDR-17010466.

We will share individual patient data (IPD) within 2 years after the trial complete, and the original data is collected by clinical recording formula (both paper and electronic version). The results will be presented at international spine related meeting

For peer review only - http://bmjopen.bmj.com/site/about/guidelines.xhtml

and peer-reviewed journals.

Reference

1. Wang JC, Dailey AT, Mummaneni PV, et al. Guideline update for the performance of fusion procedures for degenerative disease of the lumbar spine. Part 8: lumbar fusion for disc herniation and radiculopathy. *J Neurosurg Spine* 2014;21(1):48-53.
2. Brayda-Bruno M, Tibiletti M, Ito K, et al. Advances in the diagnosis of degenerated lumbar discs and their possible clinical application. *Eur Spine J* 2014;23 Suppl 3:S315-23.
3. Manchikanti L, Pampati V, Hirsch JA. Retrospective cohort study of usage patterns of epidural injections for spinal pain in the US fee-for-service Medicare population from 2000 to 2014. *BMJ Open* 2016;6(12):e013042.
4. Deyo RA, Mirza SK, Martin BI, et al. Trends, major medical complications, and charges associated with surgery for lumbar spinal stenosis in older adults. *JAMA* 2010;303(13):1259-65.
5. Sivasubramaniam V, Patel HC, Ozdemir BA, et al. Trends in hospital admissions and surgical procedures for degenerative lumbar spine disease in England: a 15-year time-series study. *BMJ Open* 2015;5(12):e009011.
6. Lurie J, Tomkins-Lane C. Management of lumbar spinal stenosis. *BMJ* 2016;352:h6234.
7. Omidi-Kashani F, Hasankhani EG, Ashjazadeh A. Lumbar spinal stenosis: who should be fused? An updated review. *Asian Spine J* 2014;8(4):521-30.
8. Wu AM, Tong TJ, Wang XY. A rethink of fusion surgery for lumbar spinal stenosis. *J Evid Based Med* 2016
9. Katz JN, Harris MB. Clinical practice. Lumbar spinal stenosis. *N Engl J Med* 2008;358(8):818-25.
10. Amoretti N, Amoretti ME, Hovorka I, et al. Percutaneous facet screw fixation of lumbar spine with CT and fluoroscopic guidance: a feasibility study. *Radiology* 2013;268(2):548-55.
11. Liu F, Jiang C, Cao Y, et al. Transforaminal lumbar interbody fusion using unilateral pedicle screw fixation plus contralateral translaminar facet screw fixation in lumbar degenerative diseases. *Indian J Orthop* 2014;48(4):374-9.
12. Luo P, Shao RX, Wu AM, et al. Transforaminal Lumbar Interbody Fusion with Unilateral Pedicle Screw and Contralateral Percutaneous Transfacet Screw Fixation for the Treatment of Lumbar Degenerative Disorders. *Turk Neurosurg* 2016;26(5):763-70.
13. Magerl FP. Stabilization of the lower thoracic and lumbar spine with external skeletal fixation. *Clin Orthop Relat Res* 1984(189):125-41.
14. Ferrara LA, Secor JL, Jin BH, et al. A biomechanical comparison of facet screw fixation and pedicle screw fixation: effects of short-term and long-term repetitive cycling. *Spine (Phila Pa 1976)* 2003;28(12):1226-34.
15. Deguchi M, Cheng BC, Sato K, et al. Biomechanical evaluation of translaminar facet joint fixation. A comparative study of poly-L-lactide pins, screws, and pedicle fixation. *Spine (Phila Pa 1976)* 1998;23(12):1307-12; discussion 13.
16. Park SH, Park WM, Park CW, et al. Minimally invasive anterior lumbar interbody fusion followed by percutaneous translaminar facet screw fixation in elderly patients. *J Neurosurg Spine* 2009;10(6):610-6.

For peer review only - http://bmjopen.bmj.com/site/about/guidelines.xhtml

17. Zeng ZY, Wu P, Song YX, et al. [Unilateral pedicle screw fixation combined with contralateral percutaneous translaminar facet screw fixation and lumbar interbody fusion for the treatment of lower lumbar diseases: an analysis of complications]. *Zhongguo Gu Shang* 2016;29(3):232-41.

18. Zeng ZY, Wu P, Yan WF, et al. Mixed Fixation and Interbody Fusion for Treatment Single-Segment Lower Lumbar Vertebral Disease: Midterm Follow-up Results. *Orthop Surg* 2015;7(4):324-32.

19. Chan AW, Tetzlaff JM, Gotzsche PC, et al. SPIRIT 2013 explanation and elaboration: guidance for protocols of clinical trials. *BMJ* 2013;346:e7586.

20. Chan AW, Tetzlaff JM, Altman DG, et al. SPIRIT 2013 statement: defining standard protocol items for clinical trials. *Ann Intern Med* 2013;158(3):200-7.

21. Shim CS, Lee SH, Jung B, et al. Fluoroscopically assisted percutaneous translaminar facet screw fixation following anterior lumbar interbody fusion: technical report. *Spine (Phila Pa 1976)* 2005;30(7):838-43.

22. Lu J, Ebraheim NA, Yeasting RA. Translaminar facet screw placement: an anatomic study. *Am J Orthop (Belle Mead NJ)* 1998;27(8):550-5.

23. Kornblatt MD, Casey MP, Jacobs RR. Internal fixation in lumbosacral spine fusion. A biomechanical and clinical study. *Clin Orthop Relat Res* 1986(203):141-50.

24. Lieberman IH, Togawa D, Kayanja MM, et al. Bone-mounted miniature robotic guidance for pedicle screw and translaminar facet screw placement: Part I--Technical development and a test case result. *Neurosurgery* 2006;59(3):641-50; discussion 41-50.

25. Wu AM, Wang S, Weng WQ, et al. The Radiological Feature of Anterior Occiput-to-Axis Screw Fixation as it Guides the Screw Trajectory on 3D Printed Models: A Feasibility Study on 3D Images and 3D Printed Models. *Medicine (Baltimore)* 2014;93(28):e242.

26. Wu AM, Shao ZX, Wang JS, et al. The accuracy of a method for printing three-dimensional spinal models. *PLoS One* 2015;10(4):e0124291.

27. Maruthappu M, Keogh B. How might 3D printing affect clinical practice. *BMJ* 2014;349

28. Sugawara T, Higashiyama N, Kaneyama S, et al. Accurate and Simple Screw Insertion Procedure with Patient-Specific Screw Guide Templates for Posterior C1-C2 Fixation. *Spine (Phila Pa 1976)* 2016

29. Liu K, Zhang Q, Li X, et al. Preliminary application of a multi-level 3D printing drill guide template for pedicle screw placement in severe and rigid scoliosis. *Eur Spine J* 2016

For peer review only

For peer review only - http://bmjopen.bmj.com/site/about/guidelines.xhtml

Table 1: The data need to collect through the research.

	Baseline	Peri-operation	Post-Operation				
Assessment	−1 day	day 0	Day 1	3 mons	6 mons	12 mons	24 mons
Eligibility criteria	×						
Recruitment	×						
Informed consent	×						
Randomization	×						
Baseline demographics	×						
Medical history	×						
X-ray exposure		×					
Wound incision length		×					
operative time		×					
Blood loss		×					
Complications		×	×	×	×	×	×
Screw angles			×				
The position of the screws			×				
VAS of back pain	×		×	×	×	×	×
VAS of leg pain	×		×	×	×	×	×
ODI	×		×	×	×	×	×

Note: VAS of back pain: Visual Analog Scale of back pain; VAS of leg pain: Visual Analog Scale of leg pain; ODI: Oswestry Disability Index

For peer review only

Figure Legends

Figure 1: The flow chart of randomized controlled trial.

Figure 2:The designed pedicle screw trajectory and translaminar facet screw trajectory: α is the angle between the translaminar facet screw trajectory and the vertical line in the anteroposterior view; β is the angle between translaminar facet screw trajectory and the upper endplate of lower vertebra in the lateral view; γ is the angle between translaminar facet screw trajectory and midline of the lumbar vertebra in the top view; δ and ε are the angles between pedicle screw trajectory (upper vertebra and lower vertebra) and midline of the lumbar vertebra in the top view.

Figure 3: The designed 3D navigation template in CATIA software.

Figure 4: 3D navigation template imported into MeditoolCreate software to print.

For peer review only

For peer review only - http://bmjopen.bmj.com/site/about/guidelines.xhtml

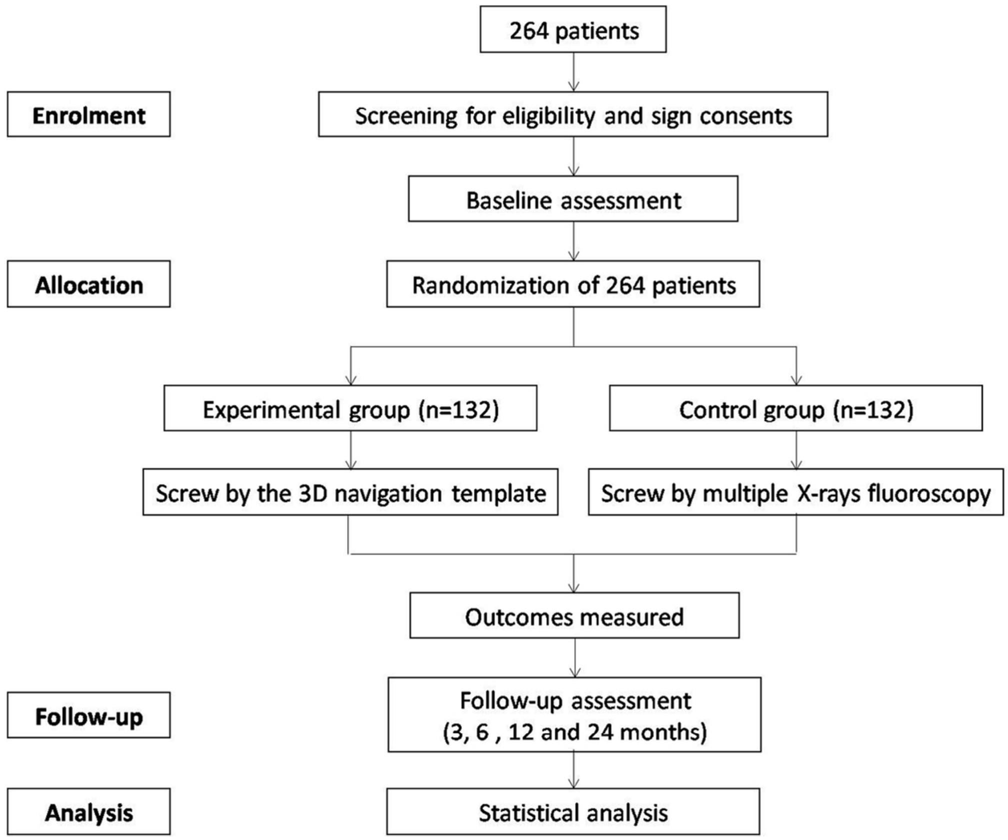

The flow chart of randomized controlled trial.

99x82mm (300 x 300 DPI)

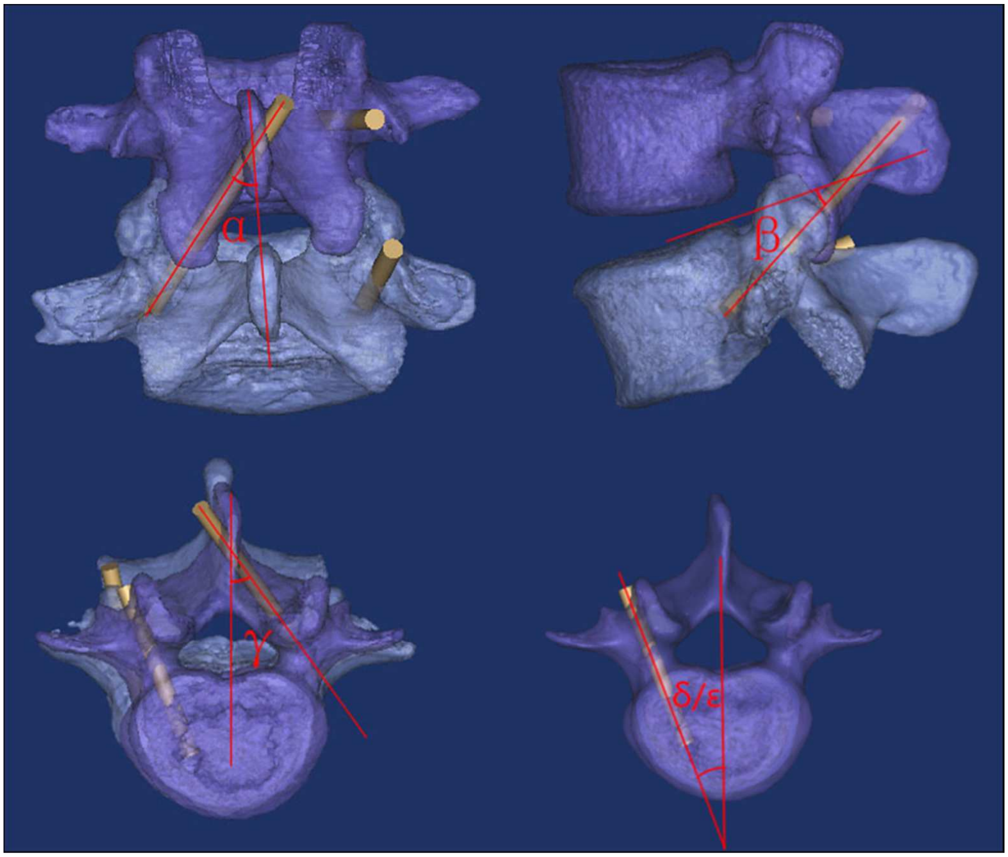

The designed pedicle screw trajectory and translaminar facet screw trajectory: α is the angle between the translaminar facet screw trajectory and the vertical line in the anteroposterior view; β is the angle between translaminar facet screw trajectory and the upper endplate of lower vertebra in the lateral view; γ is the angle between translaminar facet screw trajectory and midline of the lumbar vertebra in the top view; δ and ε are the angles between pedicle screw trajectory (upper vertebra and lower vertebra) and midline of the lumbar vertebra in the top view.

101x86mm (300 x 300 DPI)

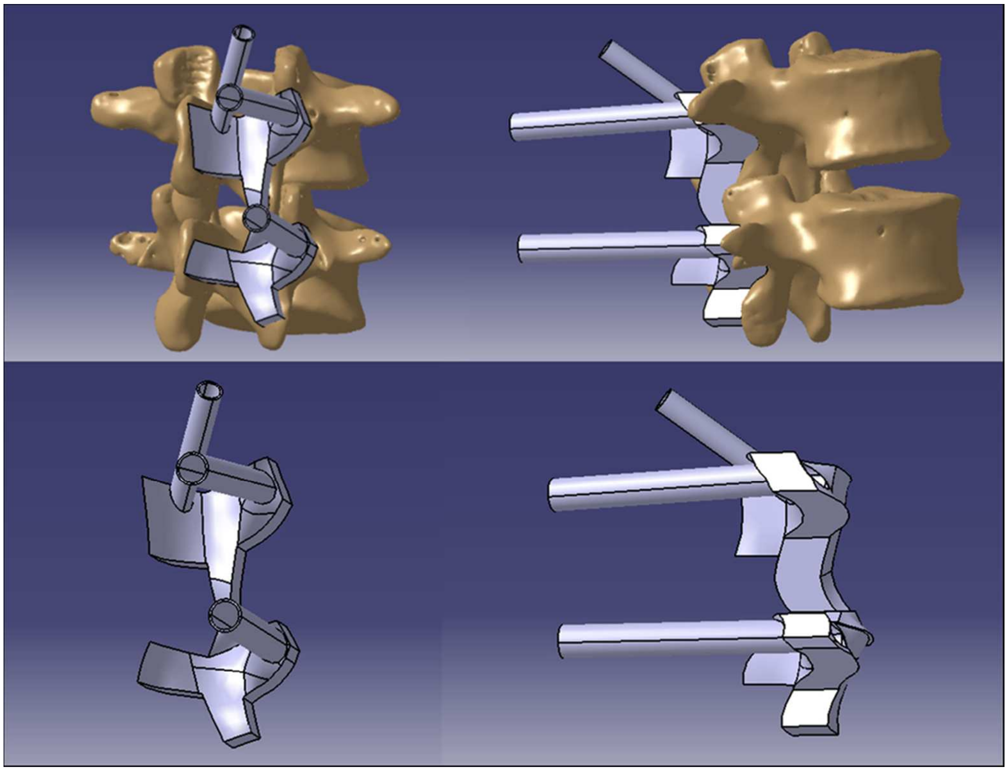

The designed 3D navigation template in CATIA software.

91x69mm (300 x 300 DPI)

For peer review only

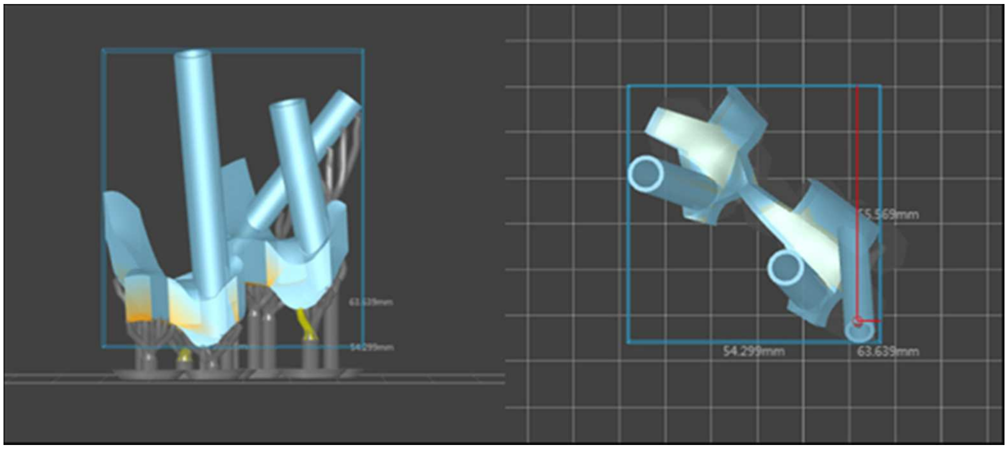

3D navigation template imported into MeditoolCreate software to print.

52x23mm (300 x 300 DPI)

For peer review only

SPIRIT

STANDARD PROTOCOL ITEMS: RECOMMENDATIONS FOR INTERVENTIONAL TRIALS

SPIRIT 2013 Checklist: Recommended items to address in a clinical trial protocol and related documents*

Section/item	Item No	Description	Addressed on page number
**Administrative information**			
Title	1	Descriptive title identifying the study design, population, interventions, and, if applicable, trial acronym	___3__________
Trial registration	2a	Trial identifier and registry name. If not yet registered, name of intended registry	___4__________
	2b	All items from the World Health Organization Trial Registration Data Set	___6__________
Protocol version	3	Date and version identifier	___2__________
Funding	4	Sources and types of financial, material, and other support	___2__________
Roles and responsibilities	5a	Names, affiliations, and roles of protocol contributors	___1__________
	5b	Name and contact information for the trial sponsor	___2__________
	5c	Role of study sponsor and funders, if any, in study design; collection, management, analysis, and interpretation of data; writing of the report; and the decision to submit the report for publication, including whether they will have ultimate authority over any of these activities	___2__________
	5d	Composition, roles, and responsibilities of the coordinating centre, steering committee, endpoint adjudication committee, data management team, and other individuals or groups overseeing the trial, if applicable (see Item 21a for data monitoring committee)	___2__________

For peer review only

For peer review only - http://bmjopen.bmj.com/site/about/guidelines.xhtml

**Introduction**			
Background and rationale	6a	Description of research question and justification for undertaking the trial, including summary of relevant studies (published and unpublished) examining benefits and harms for each intervention	___5__________
	6b	Explanation for choice of comparators	__5___________
Objectives	7	Specific objectives or hypotheses	__6___________
Trial design	8	Description of trial design including type of trial (eg, parallel group, crossover, factorial, single group), allocation ratio, and framework (eg, superiority, equivalence, noninferiority, exploratory)	__6___________
**Methods: Participants, interventions, and outcomes**			
Study setting	9	Description of study settings (eg, community clinic, academic hospital) and list of countries where data will be collected. Reference to where list of study sites can be obtained	__6___________
Eligibility criteria	10	Inclusion and exclusion criteria for participants. If applicable, eligibility criteria for study centres and individuals who will perform the interventions (eg, surgeons, psychotherapists)	_7___________
Interventions	11a	Interventions for each group with sufficient detail to allow replication, including how and when they will be administered	__6 and Figure 1
	11b	Criteria for discontinuing or modifying allocated interventions for a given trial participant (eg, drug dose change in response to harms, participant request, or improving/worsening disease)	__7___________
	11c	Strategies to improve adherence to intervention protocols, and any procedures for monitoring adherence (eg, drug tablet return, laboratory tests)	__8-9_________
	11d	Relevant concomitant care and interventions that are permitted or prohibited during the trial	__9__________
Outcomes	12	Primary, secondary, and other outcomes, including the specific measurement variable (eg, systolic blood pressure), analysis metric (eg, change from baseline, final value, time to event), method of aggregation (eg, median, proportion), and time point for each outcome. Explanation of the clinical relevance of chosen efficacy and harm outcomes is strongly recommended	__11_________
Participant timeline	13	Time schedule of enrolment, interventions (including any run-ins and washouts), assessments, and visits for participants. A schematic diagram is highly recommended (see Figure)	__11 and table 1_

For peer review only - http://bmjopen.bmj.com/site/about/guidelines.xhtml

Sample size	14	Estimated number of participants needed to achieve study objectives and how it was determined, including clinical and statistical assumptions supporting any sample size calculations	___8________
Recruitment	15	Strategies for achieving adequate participant enrolment to reach target sample size	___8-9_________

Methods: Assignment of interventions (for controlled trials)

Allocation:

Sequence generation	16a	Method of generating the allocation sequence (eg, computer-generated random numbers), and list of any factors for stratification. To reduce predictability of a random sequence, details of any planned restriction (eg, blocking) should be provided in a separate document that is unavailable to those who enrol participants or assign interventions	___8_________
Allocation concealment mechanism	16b	Mechanism of implementing the allocation sequence (eg, central telephone; sequentially numbered, opaque, sealed envelopes), describing any steps to conceal the sequence until interventions are assigned	___8_________
Implementation	16c	Who will generate the allocation sequence, who will enrol participants, and who will assign participants to interventions	__8________
Blinding (masking)	17a	Who will be blinded after assignment to interventions (eg, trial participants, care providers, outcome assessors, data analysts), and how	__8_________
	17b	If blinded, circumstances under which unblinding is permissible, and procedure for revealing a participant's allocated intervention during the trial	__8___________

Methods: Data collection, management, and analysis

Data collection methods	18a	Plans for assessment and collection of outcome, baseline, and other trial data, including any related processes to promote data quality (eg, duplicate measurements, training of assessors) and a description of study instruments (eg, questionnaires, laboratory tests) along with their reliability and validity, if known. Reference to where data collection forms can be found, if not in the protocol	__10-11_______
	18b	Plans to promote participant retention and complete follow-up, including list of any outcome data to be collected for participants who discontinue or deviate from intervention protocols	__10-11________

For peer review only - http://bmjopen.bmj.com/site/about/guidelines.xhtml

Data management	19	Plans for data entry, coding, security, and storage, including any related processes to promote data quality (eg, double data entry; range checks for data values). Reference to where details of data management procedures can be found, if not in the protocol	____11________
Statistical methods	20a	Statistical methods for analysing primary and secondary outcomes. Reference to where other details of the statistical analysis plan can be found, if not in the protocol	____12_______
	20b	Methods for any additional analyses (eg, subgroup and adjusted analyses)	____NA_______
	20c	Definition of analysis population relating to protocol non-adherence (eg, as randomised analysis), and any statistical methods to handle missing data (eg, multiple imputation)	___ NA _______
**Methods: Monitoring**			
Data monitoring	21a	Composition of data monitoring committee (DMC); summary of its role and reporting structure; statement of whether it is independent from the sponsor and competing interests; and reference to where further details about its charter can be found, if not in the protocol. Alternatively, an explanation of why a DMC is not needed	___8-9_________
	21b	Description of any interim analyses and stopping guidelines, including who will have access to these interim results and make the final decision to terminate the trial	____8-9______
Harms	22	Plans for collecting, assessing, reporting, and managing solicited and spontaneously reported adverse events and other unintended effects of trial interventions or trial conduct	____8-9______
Auditing	23	Frequency and procedures for auditing trial conduct, if any, and whether the process will be independent from investigators and the sponsor	____8-9_______
**Ethics and dissemination**			
Research ethics approval	24	Plans for seeking research ethics committee/institutional review board (REC/IRB) approval	____13______
Protocol amendments	25	Plans for communicating important protocol modifications (eg, changes to eligibility criteria, outcomes, analyses) to relevant parties (eg, investigators, REC/IRBs, trial participants, trial registries, journals, regulators)	__13_______

For peer review only - http://bmjopen.bmj.com/site/about/guidelines.xhtml

Consent or assent	26a	Who will obtain informed consent or assent from potential trial participants or authorised surrogates, and how (see Item 32)	_____13_______
	26b	Additional consent provisions for collection and use of participant data and biological specimens in ancillary studies, if applicable	___13________
Confidentiality	27	How personal information about potential and enrolled participants will be collected, shared, and maintained in order to protect confidentiality before, during, and after the trial	___13________
Declaration of interests	28	Financial and other competing interests for principal investigators for the overall trial and each study site	___2________
Access to data	29	Statement of who will have access to the final trial dataset, and disclosure of contractual agreements that limit such access for investigators	______________
Ancillary and post-trial care	30	Provisions, if any, for ancillary and post-trial care, and for compensation to those who suffer harm from trial participation	___13________
Dissemination policy	31a	Plans for investigators and sponsor to communicate trial results to participants, healthcare professionals, the public, and other relevant groups (eg, via publication, reporting in results databases, or other data sharing arrangements), including any publication restrictions	___13_________
	31b	Authorship eligibility guidelines and any intended use of professional writers	___13___________
	31c	Plans, if any, for granting public access to the full protocol, participant-level dataset, and statistical code	____13_________
**Appendices**			
Informed consent materials	32	Model consent form and other related documentation given to participants and authorised surrogates	_____NA_______
Biological specimens	33	Plans for collection, laboratory evaluation, and storage of biological specimens for genetic or molecular analysis in the current trial and for future use in ancillary studies, if applicable	______NA____

*It is strongly recommended that this checklist be read in conjunction with the SPIRIT 2013 Explanation & Elaboration for important clarification on the items. Amendments to the protocol should be tracked and dated. The SPIRIT checklist is copyrighted by the SPIRIT Group under the Creative Commons "Attribution-NonCommercial-NoDerivs 3.0 Unported" license.

For peer review only - http://bmjopen.bmj.com/site/about/guidelines.xhtml

BMJ Open

A 3D navigation template for guiding a unilateral lumbar pedicle screw with contralateral translaminar facet screw fixation: A study protocol for multicentre randomized controlled trials.

Journal:	*BMJ Open*
Manuscript ID	bmjopen-2017-016328.R1
Article Type:	Protocol
Date Submitted by the Author:	11-May-2017
Complete List of Authors:	Shao, Zhen-Xuan; Orthopaedic Hospital, The Second Affiliated Hospital and Yuying Children's Hospital of the Wenzhou Medical University, The Second Medical School of the Wenzhou Medical University, Zhejiang Spine Center, Wenzhou, China., Department of Spine Surgery<br>He, Wei; People´s Hospital of Shaoxing, Zhejiang University Shaoxing Hospital, Shaoxing, China., Department of Orthopaedics<br>He, Shao-Qi; People´s Hospital of Shaoxing, Zhejiang University Shaoxing Hospital, Shaoxing, Zhejiang, China., Department of Orthopaedics<br>Lin, Sheng-Lei; Wenzhou Center Hospital, Dingli Hospital of Wenzhou Medical University, Wenzhou, China., Department of Orthopaedics<br>Huang, Zhe-Yu; Ningbo No. 6 Hospital, Ningbo, Zhejiang, China, Department of Orthopaedics<br>Tang, Hong-Chao; Jiaxing Hospital of Zhejiang General Corps of Armed Police Forces, Jiaxing, China, Department of Orthopaedics<br>ni, wenfei<br>Wang, Xiang-Yang; Second Affiliated Hospital of Wenzhou Medical University, Second Medical College of Wenzhou Medical University<br>Wu, Ai-Min; Orthopedics
**Primary Subject Heading**:	Surgery
Secondary Subject Heading:	Evidence based practice
Keywords:	3D navigation template, Translaminar facet screw fixation, Multicenter randomized controlled trials, Study protocol

SCHOLARONE™
Manuscripts

Title: A 3D navigation template for guiding a unilateral lumbar pedicle screw with contralateral translaminar facet screw fixation: A study protocol for multicentre randomized controlled trials.

Authors: Zhen-Xuan Shao[1] MD; Wei-He[2] MD; Shao-Qi He[3] MD; Sheng-Lei Lin[4] MD; Zhe-Yu Huang[5] MD; Hong-Chao Tang MD[6], Wen-Fei Ni[1] MD PhD; Xiang-Yang Wang[1] MD PhD; Ai-Min Wu[1]* MD PhD;

Affiliated:

1. Department of Spine Surgery, Orthopaedic Hospital, The Second Affiliated Hospital and Yuying Children's Hospital of the Wenzhou Medical University, The Second Medical School of the Wenzhou Medical University, Zhejiang Spine Center, Wenzhou, China.

2. Department of Orthopaedics, People´s Hospital of Shaoxing, Zhejiang University Shaoxing Hospital, Shaoxing, China.

3. Department of Orthopaedics, People's Hospital of Ruian, The Third Affiliated Hospital of Wenzhou Medical University, Ruian, China.

4. Department of Orthopaedics, Wenzhou Center Hospital, Dingli Hospital of Wenzhou Medical University, Wenzhou, China.

5. Department of Orthopaedics, Ningbo No. 6 Hospital, Ningbo, Zhejiang, China.

6. Department of Orthopaedics, Jiaxing Hospital of Zhejiang General Corps of Armed Police Forces, Jiaxing, China.

For peer review only

For peer review only - http://bmjopen.bmj.com/site/about/guidelines.xhtml

Funds and Acknowledgement: This work was funded by the National Natural Science Foundation of China (81501933), Zhejiang science and technology innovation project (2016R413061), Wenzhou Science and Technology Project (Y20160369). The funders had no role in the design, execution, or writing of the study.

Disclosures: All authors declare that they have no conflict of interest.

Author contributors: XYW and AMW is the principal investigator of this trial. ZXS, WFN, XYW, and AMW contributed to the development of the research question and trial design. ZXS and AMW contributed to the drafting of the manuscript, ZXS, WH, SQH, SLL, ZYH and HCT are responsible for the recruitment and treatment of patients. All authors edited the final manuscript. All authors read and approved the final manuscript.

***Corresponding author:**

Ai-Min Wu MD PhD E-mail: aiminwu2005@163.com

Affiliated:

Department of Spine Surgery, Orthopaedic Hospital, The Second Affiliated Hospital and Yuying Children's Hospital of the Wenzhou Medical University, The Second Medical School of the Wenzhou Medical University, Zhejiang Spine Center, Wenzhou, China.

Address: 109# Xueyuan Xi Road, Wenzhou, China

Postal code: 325027

For peer review only

For peer review only - http://bmjopen.bmj.com/site/about/guidelines.xhtml

Phone: 8613758745616

Fax: 8657788002823

Keywords: 3D navigation template; Translaminar facet screw fixation; Multicentre randomized controlled trials; Study protocol.

Word count - 2635

For peer review only

For peer review only - http://bmjopen.bmj.com/site/about/guidelines.xhtml

A 3D navigation template for guiding a unilateral lumbar pedicle screw with contralateral translaminar facet screw fixation: A study protocol for multicentre randomized controlled trials.

Abstract

Introduction: The incidence of lumbar disc degeneration disease has increased in recent years. Lumbar interbody fusion using two unilateral pedicle screws and a translaminar facet screw fixation has advantages of minimal invasiveness and lower costs compared with the traditional methods. Moreover, a method guided by a 3D navigation template may help us improve the surgical accuracy and the success rate. This is the first randomized study using a 3D navigation template to guide a unilateral lumbar pedicle screw with contralateral translaminar facet screw fixation.

Methods and Analysis: Patients who meet the criteria of the surgery will be randomly divided into experimental groups and control groups by a computer-generated randomization schedule. We will preoperatively design an individual 3D navigation template using CATIA software and MeditoolCreate. The following primary outcomes will be collected: screw angles compared with the optimal screw trajectories in 3D digital images; length of the wound incision; operative time; intra-operative blood loss; and complications. The following secondary outcomes will be collected: Visual Analogue Scale for back pain (VAS for back pain); Visual Analogue Scale for leg pain (VAS for leg pain); and the Oswestry Disability Index (ODI). These parameters will be evaluated on day 1 and then 3, 6, 12

For peer review only

and 24 months postoperatively.

Ethics and dissemination: The study has been reviewed and approved by the IRB of the Second Affiliated Hospital and Yuying Children's Hospital of Wenzhou Medical University. The results will be presented at scientific communities and peer-reviewed journals.

Trial registration number: ChiCTR-IDR-17010466

Keywords: 3D navigation template; Translaminar facet screw fixation; Multicentre randomized controlled trials; Study protocol.

Strengths and limitations of this study

1. This is the first multicentre randomized controlled trial to compare a 3D navigation template for guiding a unilateral lumbar pedicle screw with contralateral translaminar facet screw fixation vs. traditional multiple X-ray fluoroscopy.

2. We will first use Mimics and CATIA software to design the 3D navigation template of a unilateral lumbar pedicle screw with contralateral translaminar facet screw fixation.

3. The clinical data will be collected prospectively at least 24 months after the operation.

4. Because this is a multicentre randomized controlled trial, the surgeons' experiments at different research sites may influence the outcomes.

For peer review only - http://bmjopen.bmj.com/site/about/guidelines.xhtml

Introduction

With the ageing of society, the incidence of lumbar disc degeneration disease is increasing [1 2], which is a major socioeconomic burden [3-5]. If conservative treatment of lumbar degenerative disease is ineffective, surgical intervention is recommended for these patients [6-9]. Compared to the pedicle screw fixation on both sides, which has gained popularity among general surgeons because of easiness, the lumbar interbody fusion, which uses two unilateral pedicle screws and a translaminar facet screw fixation, can reduce tissue trauma and the length of the wound incision, as well as promote recovery[10-12].

The translaminar screw is a long screw that goes through the spinous process and zygapophyseal joints and ends in a lower vertebral facet to fix the other side of the joint[13]. The biomechanical properties of the translaminar screw are the same as pedicle screws in vitro biomechanical experiments[14 15]. Park et al[16] reported that the VAS and ODI scores of the patient were significantly improved after minimally invasive surgery of anterior lumbar interbody fusion combined with the translaminar screw. Liu et al [11] reported a recovery rate of 93.5% through minimally invasive surgery of transforaminal lumbar interbody fusion combined with the translaminar screw.

However, there is higher risk of nerve and vascular injury when the angle deviation of the translaminar screw or screw entry point is incorrect[17]. To achieve an optimal screw trajectory, many surgeons choose multiple X-ray images as a guide to locate the entrance point and adjust the angle, which will expose both patients and surgeons to

For peer review only - http://bmjopen.bmj.com/site/about/guidelines.xhtml

For peer review only

considerable radiation[11 12 18]. The technique of 3D reconstruction and a 3D rapid printed navigation template, which extracts the posterior surface features of the lumbar vertebrae, may help determine the optimal trajectory of translaminar facet screw fixation.

To the best of our knowledge, there are no randomized controlled studies of a 3D navigation template vs. traditional multiple X-ray fluoroscopy for guiding a unilateral lumbar pedicle screw with contralateral translaminar facet screw fixation. In this study, we will conduct a randomized controlled trial (RCT) to compare 3D navigation template for guiding a unilateral lumbar pedicle screw with contralateral translaminar facet screw fixation vs. traditional multiple X-ray fluoroscopy.

Methods and Analysis

The study has been reviewed and approved by the Institutional Ethics Review Board of the Second Affiliated Hospital and Yuying Children's Hospital of Wenzhou Medical University. All of the participants will sign their informed consent. The protocol has been registered in the Chinese Clinical Trial Registry (ChiCTR), assigned to be the representative registry of China to join the WHO ICTRP in 2007, with protocol number ChiCTR-IDR-17010466. The 2013 SPIRIT checklist (**Supplementary Table S1**) was used to check our reports[19 20].

Participants

This study is a parallel group RCT conducted at the Orthopaedic Hospital, Second

For peer review only - http://bmjopen.bmj.com/site/about/guidelines.xhtml

Affiliated Hospital and Yuying Children's Hospital of Wenzhou Medical University.

Inclusion criteria:

1. Adult patients older than 18 years;

2. Lower back pain that is chronic or combined with neurological symptoms of the lower extremities;

3. Single-segment lower lumbar vertebral disease (including lumbar spinal canal stenosis, foraminal stenosis, segmental instability, lumbar disc herniation, and painful disc degeneration [back disc]);

4. Inefficacy after strict conservative treatment for more than 6 months.

Exclusion criteria:

1. Serious deformity of the lumbar vertebrae;

2. Dysplasia of the lumbar pedicle or vertebral lamina;

3. Obvious osteoporosis of the lumbar vertebrae;

4. Metabolic bone diseases such as osteomalacia or Paget's disease;

5. Spondylolisthesis>grade 2 (Meyerding);

6. Cauda equina injury or severe radiculopathy;

7. Post inflammatory instability of the vertebral spine;

8. Body mass index >30;

9. Immunologic diseases or metabolic syndrome;

10. Therapy with systematic corticosteroids or immunosuppressants;

11. Current use of Coumadin (warfarin) or heparin therapy for more than 6 months at

For peer review only - http://bmjopen.bmj.com/site/about/guidelines.xhtml

the time of the operation.

Sample size calculation

We performed a power analysis to assess the required sample size to show safety with a power (1-b) of 0.8 and α of 0.05. Based on the pre-experiment and related literature[21], the proportion of the control group was set to 84.6%, and the proportion of intervention group was set to 95%. We performed a Two Independent Proportions Power Analysis on PASS (Power Analysis and Sample Size), and the result was 132.

Randomization and blinding

Patients will be randomly divided into an experimental group and a control group using a computer-generated randomization schedule. The random sequence will be stored in a sealed opaque envelope to ensure that the patients, personnel and the outcome assessor will be blinded to the group allocation. Until the last questionnaires have been completed, the participant’s allocated intervention will be revealed by the study secretary. If a patient's condition deteriorates, the blinding experiment will be terminated, and the patient’s safety will be given the highest priority (**Figure 1**).

Monitoring

After screening, patients meeting the inclusion criteria should participate in the study as long as possible. If not, we must save the record to judge bias. Meanwhile, recording the patient's basic information is necessary to discuss the results of the

For peer review only - http://bmjopen.bmj.com/site/about/guidelines.xhtml

experiment.

Before the experiment, we will explain the purpose of the experiment and the importance of the experimental procedures, to obtain consent and ideal compliance. If the selected patient is unqualified, elimination will be undesirable, and the data of the patient will be substituted by the average of the group. Furthermore, loss to follow-up is inevitable; the data of these patients will also be replaced by the average.

Surgeons and data collectors will undergo training, and a preliminary experiment involving the data measurement and questionnaire will be conducted in advance. Recording the time and staff is necessary when the collector gathers the data, which will be convenient to review.

Study procedure

A retrospective review of all patients will be conducted by examining their medical records before the operation. The involved segments were identified via a C-arm machine and then marked on the patient's skin. The patients' CT images with a thickness less than 1 mm will be exported from the Star PACS image system of the hospital and imported into the Mimics 16.0 to construct 3D images, in which we adjust the threshold depending on the bone density. We regard the two adjacent segments as a functional segmental unit (FSU), such as L2-3, L3-4, L4-5, or L5-S1.

Then, the individual 3D navigation templates will be designed and printed using CATIA software and MeditoolCreate. The detailed steps are as follows: The FSU digital data in the Mimics 16.0 will be saved in STL format and imported into CATIA

For peer review only - http://bmjopen.bmj.com/site/about/guidelines.xhtml

software. We will establish the optimal screw trajectory, which includes two vertebral pedicle screws trajectories and one contralateral translaminar facet screw trajectory. We will measure three angles of the translaminar facet screw trajectory: α, β, γ. α is the angle between the screw trajectory and the vertical line in the anteroposterior view; β is the angle between the screw trajectory and the upper endplate of the lower vertebra in the lateral view; γ is the angle between the screw trajectory and the midline of the lumbar vertebra in the top view (**Figure 2**). The pedicle screw trajectories will be designed according to method of Roy-Camille[12]. We will measure two angles, δ and ε, which is the angle between the ideal screw trajectory and the midline of the lumbar vertebra in the top view, respectively (**Figure 2**).

After the optimal screw trajectories were determined, the curved posterior surface of the lumbar vertebra around the screw entry point was extracted to create the 3D navigation template (**Figure 3**). Then, we imported the 3D navigation template file into the 3D printer to rapid print it through MeditoolCreate [13] (**Figure 4**). The experiment on 3D printed lumbar model will be conducted before clinical to make sure the accurate screw trajectory (**Figure 5**).

The 3D navigation template will be sterilized and used to guide the screw in further operations on the patients. In experimental group, the dissection of the paraspinal tissue will performed at one side to expose the part of spinous process, laminar, and facet joint; the 3D navigation template will be attached to the surface of the above bony construction. Then, the Kirschner wire (K-wire) will be inserted inside the 3D navigation template, and a trajectory for the screw will be fashioned with a cannulated

For peer review only - http://bmjopen.bmj.com/site/about/guidelines.xhtml

drill; screws will be subsequently introduced. In the control group, multiple X-ray fluoroscopy will be used, and the screw trajectory will be adjusted by intra-operative X-ray fluoroscopy.

After the operation, CT scans of the patients in both groups will be obtained, and the α1, β1, γ1 δ1 and ε1 angles will be measured corresponding to the angles of the optimal screw trajectory in 3D images. The placement and position of the screws will be divided into three types (type I, the screw does not penetrate out of the cortex; type II, the screw partially penetrates out of the cortex, but by less than 2 mm; and type III, the screw penetrates out of the cortex by more than 2 mm).

Outcome measure

Primary outcomes ***(Table 1)****:*

1. Screw angles compared with the optimal screw trajectories in the 3D digital images, and the position of the screws

2. Wound incision length, operative time, intra-operative blood loss, times of X-ray exposure and complications.

Secondary outcomes ***(Table 1)****:*

1. Visual Analogue Scale for back pain (VAS for back pain)

2. Visual Analogue Scale for leg pain (VAS for leg pain)

3. Oswestry Disability Index (ODI)

The data will be collected preoperatively; on day 1 postoperatively; and then at 3, 6, 12 and 24 months postoperatively.

For peer review only - http://bmjopen.bmj.com/site/about/guidelines.xhtml

Statistical analysis

All data will be analysed using SPSS 19.0 software. The differences in wound incision length, operative time, intra-operative blood loss, and angles between the two groups will be analysed using a two-independent-sample t-test with an α of 0.05. VASs and ODI scores at preoperation, postoperation, 3 months postoperatively, 6 months postoperatively and 12 months postoperatively will be analysed using a repeated-measures analysis of variance (ANOVA). The times of X-ray exposure and frequency of screw penetration out of the cortex will be compared using the Chi square test.

Discussion

Translaminar facet screw fixation has been reported as a minimally invasive technique[11 12 22], and biomechanical comparisons between facet screw and pedicle screw fixation have revealed biomechanical equivalence between them[14 15 23]. Unilateral lumbar pedicle screw may be used by some surgeons, however, Sethi et al[24] used in vitro human cadaveric lumbar spines to compare the biomechanical properties of bilateral pedicle screws (BPSs), unilateral pedicle screws (UPSs), UPSs and translaminar facet screws (UPSs+TLFSs), and unilateral single pedicle screws and translaminar facet screws (V construct). They found that the unilateral pedicle screw (UPS) construct was the least stable in all loading modes and that the added translaminar facet screw enhanced the stability and stiffness. Similar results were

For peer review only

obtained from a finite element analysis study[25]. The biomechanical study of Luo et al[26] revealed that a significantly larger displacement of the contralateral articular process was recorded in a model with only unilateral pedicle screws, and they reported that the unilateral pedicle screw combined with a contralateral translaminar facet screw had an instant and long-term equivalent biomechanical ability to that of the traditional bilateral pedicle screw (making it an alternative to the bilateral pedicle screw) and could be less invasive while maintaining stable and effective instrumentation.

Current computer-assisted surgery systems can achieve the accurate screw trajectory, however, the cost of computer-assisted surgery systems is considerable, and the manipulation is too complicated[27], whereas the accuracy remains dependent on multiple radiations.

Combining 3D reconstruction and 3D rapid printing is a new mode of production, which is applied in many fields because of the advantages of visualization, plasticity and rapid printing [28-30]. Moreover, 3D printing templates can help surgeons to achieve accurate screw placement for posterior cervical fixation and scoliosis [31 32].

In this study, we will compare the 3D navigation template to multiple X-ray fluoroscopy for guiding a unilateral lumbar pedicle screw with contralateral translaminar facet screw fixation. The compared results will include the peri-operative parameters (such as X-ray exposure, wound incision length, operative time, blood loss), screw angles of α1, β1, γ1, δ1, and ε1, postoperative complications, the VAS of back and leg pain, and the ODI.

For peer review only - http://bmjopen.bmj.com/site/about/guidelines.xhtml

The main strength of the study is the design as an RCT rather than an observational comparative study. An RCT has the advantage of controlling all possible variables due to the random sequence generation as opposed to observational studies, where confounding and bias may be more problematic. High-quality RCTs are generally regarded as the gold standard for studying the effectiveness of an intervention.

Because of some patients' death, relocation or other reasons, the representativeness of the sample is destroyed. Meanwhile, if some patients accept experimental treatment measures such as therapeutic drugs, the results will appear to be deviated. For these experiment biases, strict monitoring not only can reduce the loss of follow-up but can also improve patient compliance, through which bias will decrease.

Ethics and dissemination:

The study had been reviewed and approved by the Institutional Ethics Review Board of the Second Affiliated Hospital and Yuying Children's Hospital of Wenzhou Medical University. The procedure will be performed following the principles described in the Declaration of Helsinki. All of the participants will sign their informed consent. The protocol has been registered in the Chinese Clinical Trial Registry (ChiCTR), assigned to be the representative registry of China to join the WHO ICTRP in 2007, with protocol number ChiCTR-IDR-17010466.

We will share individual patient data (IPD) within 2 years after the trial is completed, and the original data will be collected using a clinical recording formula (both paper and electronic versions). The results will be presented at scientific

For peer review only - http://bmjopen.bmj.com/site/about/guidelines.xhtml

communities (such as the International Congress of the Chinese Orthopaedic Association) and peer-reviewed journals.

Contributor ship statement: XYW and AMW is the principal investigator of this trial. ZXS, WFN, XYW, and AMW contributed to the development of the research question and trial design. ZXS and AMW contributed to the drafting of the manuscript, ZXS, WH, SQH, SLL, ZYH and HCT are responsible for the recruitment and treatment of patients. All authors edited the final manuscript. All authors read and approved the final manuscript.

Competing interests: All authors declare that they have no conflict of interest.

Funding: This work was funded by the National Natural Science Foundation of China (81501933), Zhejiang science and technology innovation project (2016R413061), Wenzhou Science and Technology Project (Y20160369). The funders had no role in the design, execution, or writing of the study.

Data sharing statement: The full data set will be made available when this trial is completed and published. Application for the data to be released should be made in contact to AMW (principle investigator).

References

1. Wang JC, Dailey AT, Mummaneni PV, et al. Guideline update for the performance of fusion procedures for degenerative disease of the lumbar spine. Part 8: lumbar fusion for disc herniation and radiculopathy. *J Neurosurg Spine* 2014;21(1):48-53.

2. Brayda-Bruno M, Tibiletti M, Ito K, et al. Advances in the diagnosis of degenerated lumbar discs and

For peer review only - http://bmjopen.bmj.com/site/about/guidelines.xhtml

their possible clinical application. *Eur Spine J* 2014;23 Suppl 3:S315-23.
3. Manchikanti L, Pampati V, Hirsch JA. Retrospective cohort study of usage patterns of epidural injections for spinal pain in the US fee-for-service Medicare population from 2000 to 2014. *BMJ Open* 2016;6(12):e013042.
4. Deyo RA, Mirza SK, Martin BI, et al. Trends, major medical complications, and charges associated with surgery for lumbar spinal stenosis in older adults. *JAMA* 2010;303(13):1259-65.
5. Sivasubramaniam V, Patel HC, Ozdemir BA, et al. Trends in hospital admissions and surgical procedures for degenerative lumbar spine disease in England: a 15-year time-series study. *BMJ Open* 2015;5(12):e009011.
6. Lurie J, Tomkins-Lane C. Management of lumbar spinal stenosis. *BMJ* 2016;352:h6234.
7. Omidi-Kashani F, Hasankhani EG, Ashjazadeh A. Lumbar spinal stenosis: who should be fused? An updated review. *Asian Spine J* 2014;8(4):521-30.
8. Wu AM, Tong TJ, Wang XY. A rethink of fusion surgery for lumbar spinal stenosis. *J Evid Based Med* 2016
9. Katz JN, Harris MB. Clinical practice. Lumbar spinal stenosis. *N Engl J Med* 2008;358(8):818-25.
10. Amoretti N, Amoretti ME, Hovorka I, et al. Percutaneous facet screw fixation of lumbar spine with CT and fluoroscopic guidance: a feasibility study. *Radiology* 2013;268(2):548-55.
11. Liu F, Jiang C, Cao Y, et al. Transforaminal lumbar interbody fusion using unilateral pedicle screw fixation plus contralateral translaminar facet screw fixation in lumbar degenerative diseases. *Indian J Orthop* 2014;48(4):374-9.
12. Luo P, Shao RX, Wu AM, et al. Transforaminal Lumbar Interbody Fusion with Unilateral Pedicle Screw and Contralateral Percutaneous Transfacet Screw Fixation for the Treatment of Lumbar Degenerative Disorders. *Turk Neurosurg* 2016;26(5):763-70.
13. Magerl FP. Stabilization of the lower thoracic and lumbar spine with external skeletal fixation. *Clin Orthop Relat Res* 1984(189):125-41.
14. Ferrara LA, Secor JL, Jin BH, et al. A biomechanical comparison of facet screw fixation and pedicle screw fixation: effects of short-term and long-term repetitive cycling. *Spine (Phila Pa 1976)* 2003;28(12):1226-34.
15. Deguchi M, Cheng BC, Sato K, et al. Biomechanical evaluation of translaminar facet joint fixation. A comparative study of poly-L-lactide pins, screws, and pedicle fixation. *Spine (Phila Pa 1976)* 1998;23(12):1307-12; discussion 13.
16. Park SH, Park WM, Park CW, et al. Minimally invasive anterior lumbar interbody fusion followed by percutaneous translaminar facet screw fixation in elderly patients. *J Neurosurg Spine* 2009;10(6):610-6.
17. Zeng ZY, Wu P, Song YX, et al. [Unilateral pedicle screw fixation combined with contralateral percutaneous translaminar facet screw fixation and lumbar interbody fusion for the treatment of lower lumbar diseases: an analysis of complications]. *Zhongguo Gu Shang* 2016;29(3):232-41.
18. Zeng ZY, Wu P, Yan WF, et al. Mixed Fixation and Interbody Fusion for Treatment Single-Segment Lower Lumbar Vertebral Disease: Midterm Follow-up Results. *Orthop Surg* 2015;7(4):324-32.
19. Chan AW, Tetzlaff JM, Gotzsche PC, et al. SPIRIT 2013 explanation and elaboration: guidance for protocols of clinical trials. *BMJ* 2013;346:e7586.
20. Chan AW, Tetzlaff JM, Altman DG, et al. SPIRIT 2013 statement: defining standard protocol items for clinical trials. *Ann Intern Med* 2013;158(3):200-7.

For peer review only - http://bmjopen.bmj.com/site/about/guidelines.xhtml

21. Shim CS, Lee SH, Jung B, et al. Fluoroscopically assisted percutaneous translaminar facet screw fixation following anterior lumbar interbody fusion: technical report. *Spine (Phila Pa 1976)* 2005;30(7):838-43.

22. Lu J, Ebraheim NA, Yeasting RA. Translaminar facet screw placement: an anatomic study. *Am J Orthop (Belle Mead NJ)* 1998;27(8):550-5.

23. Kornblatt MD, Casey MP, Jacobs RR. Internal fixation in lumbosacral spine fusion. A biomechanical and clinical study. *Clin Orthop Relat Res* 1986(203):141-50.

24. Sethi A, Muzumdar AM, Ingalhalikar A, et al. Biomechanical analysis of a novel posterior construct in a transforaminal lumbar interbody fusion model an in vitro study. *Spine J* 2011;11(9):863-9.

25. Gong Z, Chen Z, Feng Z, et al. Finite element analysis of 3 posterior fixation techniques in the lumbar spine. *Orthopedics* 2014;37(5):e441-8.

26. Luo B, Yan M, Huang J, et al. Biomechanical study of unilateral pedicle screw combined with contralateral translaminar facet screw in transforaminal lumbar interbody fusion. *Clin Biomech (Bristol, Avon)* 2015;30(7):657-61.

27. Lieberman IH, Togawa D, Kayanja MM, et al. Bone-mounted miniature robotic guidance for pedicle screw and translaminar facet screw placement: Part I--Technical development and a test case result. *Neurosurgery* 2006;59(3):641-50; discussion 41-50.

28. Wu AM, Wang S, Weng WQ, et al. The Radiological Feature of Anterior Occiput-to-Axis Screw Fixation as it Guides the Screw Trajectory on 3D Printed Models: A Feasibility Study on 3D Images and 3D Printed Models. *Medicine (Baltimore)* 2014;93(28):e242.

29. Wu AM, Shao ZX, Wang JS, et al. The accuracy of a method for printing three-dimensional spinal models. *PLoS One* 2015;10(4):e0124291.

30. Maruthappu M, Keogh B. How might 3D printing affect clinical practice. *BMJ* 2014;349

31. Sugawara T, Higashiyama N, Kaneyama S, et al. Accurate and Simple Screw Insertion Procedure with Patient-Specific Screw Guide Templates for Posterior C1-C2 Fixation. *Spine (Phila Pa 1976)* 2016

32. Liu K, Zhang Q, Li X, et al. Preliminary application of a multi-level 3D printing drill guide template for pedicle screw placement in severe and rigid scoliosis. *Eur Spine J* 2016

For peer review only

For peer review only - http://bmjopen.bmj.com/site/about/guidelines.xhtml

Table 1: The data need to collect through the research.

	Baseline	Peri-operation	Post-Operation				
Assessment	−1 day	day 0	Day 1	3 mons	6 mons	12 mons	24 mons
Eligibility criteria	×						
Recruitment	×						
Informed consent	×						
Randomization	×						
Baseline demographics	×						
Medical history	×						
X-ray exposure		×					
Wound incision length		×					
operative time		×					
Blood loss		×					
Complications		×	×	×	×	×	×
Screw angles							
α1			×				
β1			×				
γ1			×				
δ1			×				
ε1			×				
The position of the screws			×				
VAS of back pain	×		×	×	×	×	×
VAS of leg pain	×		×	×	×	×	×
ODI	×		×	×	×	×	×

Note: VAS of back pain: Visual Analog Scale of back pain; VAS of leg pain: Visual Analog Scale of leg pain; ODI: Oswestry Disability Index

For peer review only

For peer review only - http://bmjopen.bmj.com/site/about/guidelines.xhtml

Figure Legends

Figure 1: The flow chart of randomized controlled trial.

Figure 2:The designed pedicle screw trajectory and translaminar facet screw trajectory: α is the angle between the translaminar facet screw trajectory and the vertical line in the anteroposterior view; β is the angle between translaminar facet screw trajectory and the upper endplate of lower vertebra in the lateral view; γ is the angle between translaminar facet screw trajectory and midline of the lumbar vertebra in the top view; δ and ε are the angles between pedicle screw trajectory (upper vertebra and lower vertebra) and midline of the lumbar vertebra in the top view.

Figure 3: The designed 3D navigation template in CATIA software.

Figure 4: 3D navigation template imported into MeditoolCreate software to print.

Figure 5: One example of the experiment on conducted 3D printed lumbar model before clinical application to make sure the accurate screw trajectory.

For peer review only

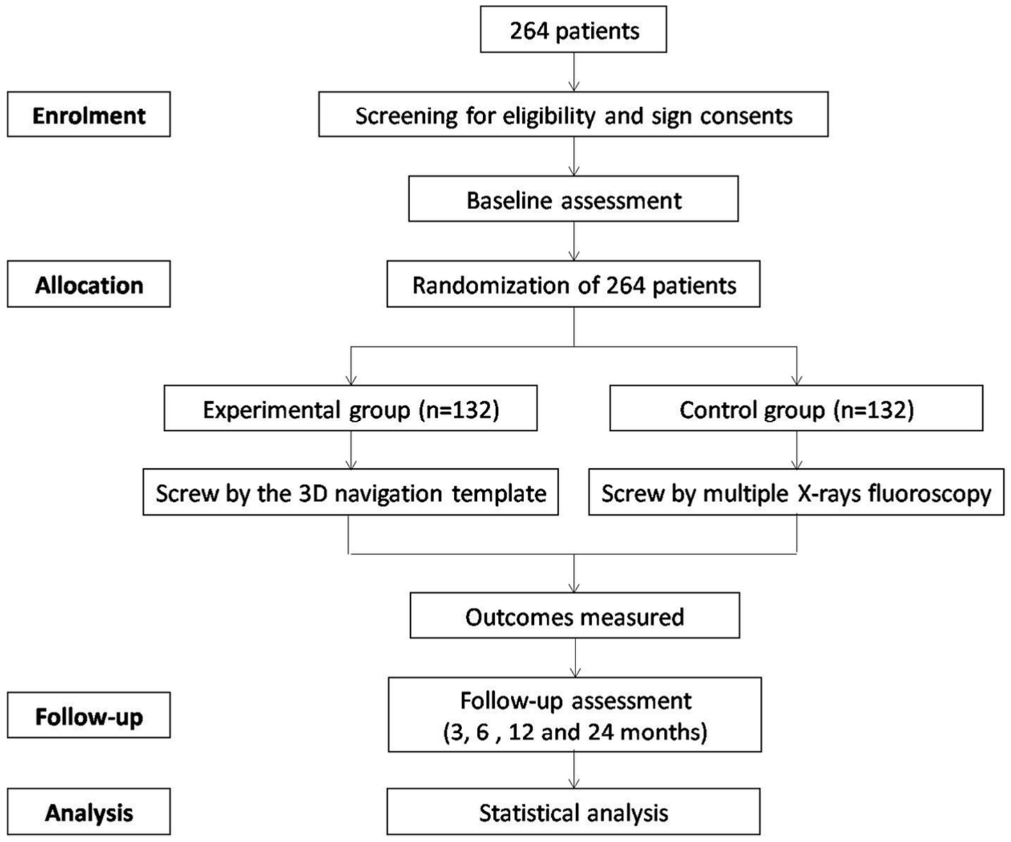

The flow chart of randomized controlled trial.

99x82mm (300 x 300 DPI)

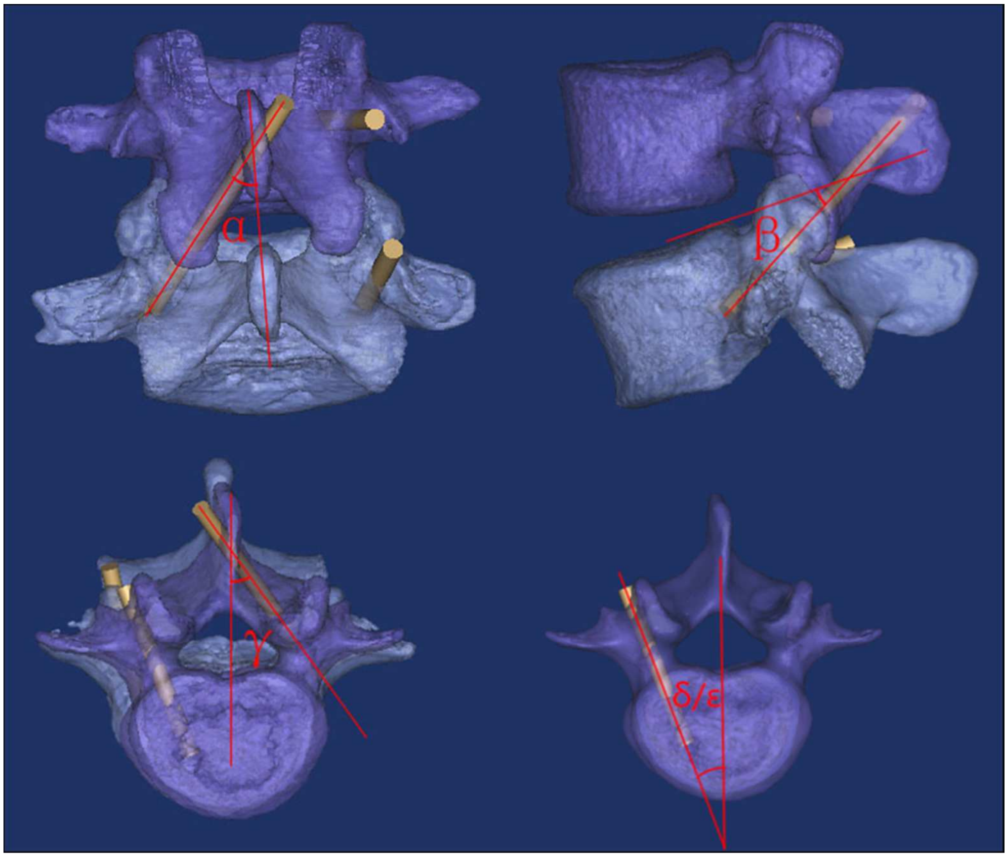

The designed pedicle screw trajectory and translaminar facet screw trajectory: α is the angle between the translaminar facet screw trajectory and the vertical line in the anteroposterior view; β is the angle between translaminar facet screw trajectory and the upper endplate of lower vertebra in the lateral view; γ is the angle between translaminar facet screw trajectory and midline of the lumbar vertebra in the top view; δ and ε are the angles between pedicle screw trajectory (upper vertebra and lower vertebra) and midline of the lumbar vertebra in the top view.

101x86mm (300 x 300 DPI)

For peer review only

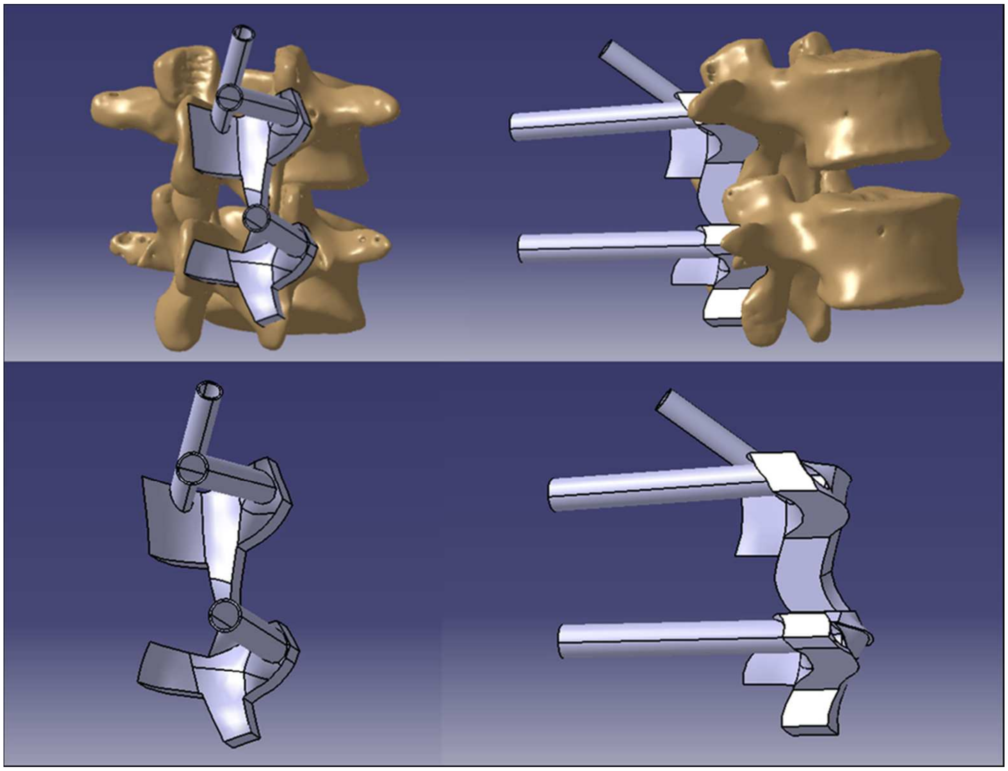

The designed 3D navigation template in CATIA software.

91x69mm (300 x 300 DPI)

For peer review only - http://bmjopen.bmj.com/site/about/guidelines.xhtml

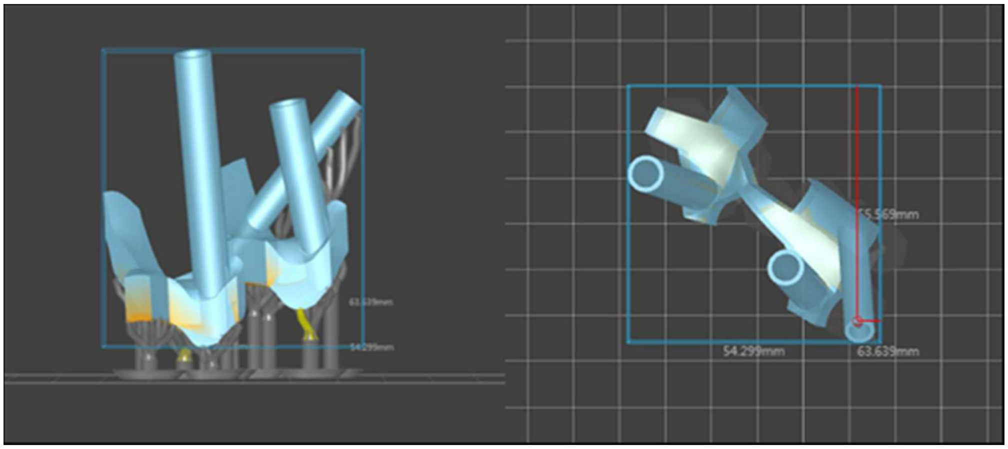

3D navigation template imported into MeditoolCreate software to print.

52x23mm (300 x 300 DPI)

er review only

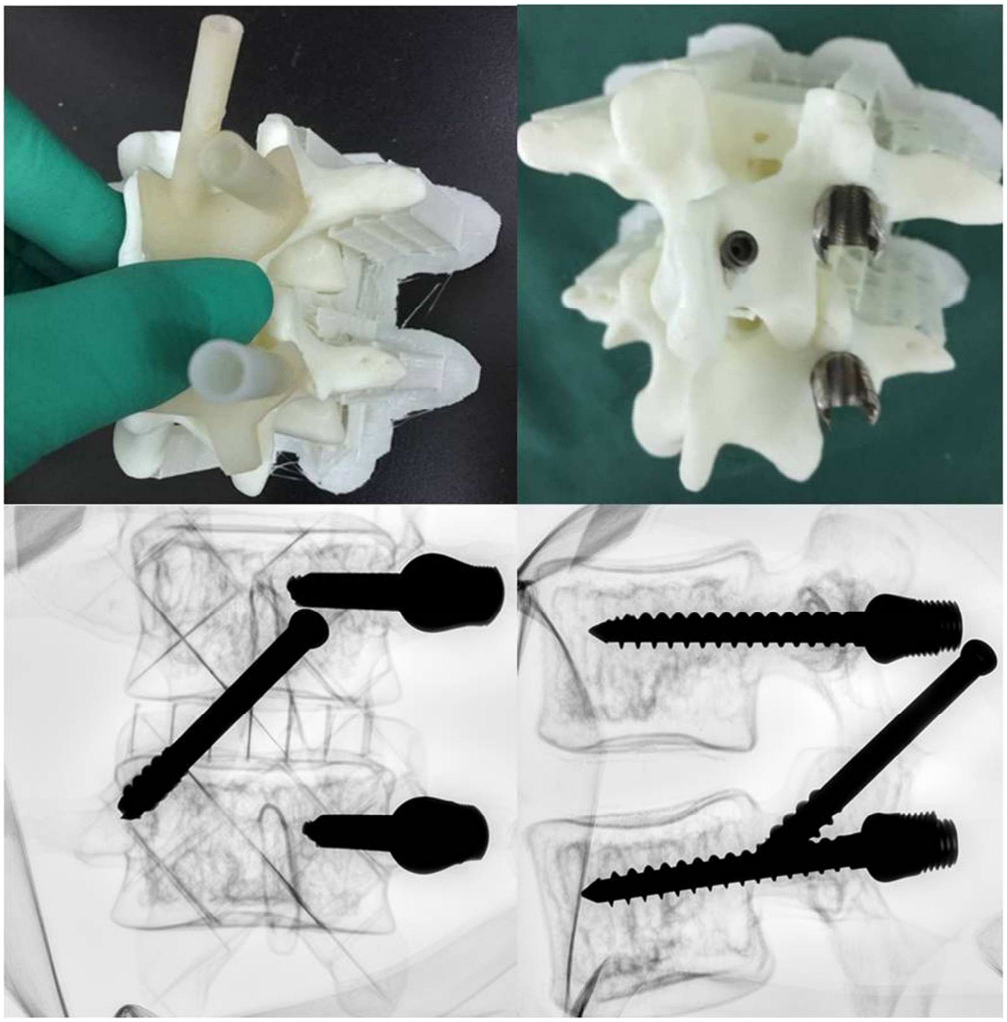

Figure 5: One example of the experiment on conducted 3D printed lumbar model before clinical application to make sure the accurate screw trajectory.

121x123mm (300 x 300 DPI)

STANDARD PROTOCOL ITEMS: RECOMMENDATIONS FOR INTERVENTIONAL TRIALS

SPIRIT 2013 Checklist: Recommended items to address in a clinical trial protocol and related documents*

Section/item	Item No	Description	Addressed on page number
**Administrative information**			
Title	1	Descriptive title identifying the study design, population, interventions, and, if applicable, trial acronym	___3___________
Trial registration	2a	Trial identifier and registry name. If not yet registered, name of intended registry	___4___________
	2b	All items from the World Health Organization Trial Registration Data Set	___6___________
Protocol version	3	Date and version identifier	___2___________
Funding	4	Sources and types of financial, material, and other support	___2___________
Roles and responsibilities	5a	Names, affiliations, and roles of protocol contributors	___1___________
	5b	Name and contact information for the trial sponsor	___2___________
	5c	Role of study sponsor and funders, if any, in study design; collection, management, analysis, and interpretation of data; writing of the report; and the decision to submit the report for publication, including whether they will have ultimate authority over any of these activities	___2___________
	5d	Composition, roles, and responsibilities of the coordinating centre, steering committee, endpoint adjudication committee, data management team, and other individuals or groups overseeing the trial, if applicable (see Item 21a for data monitoring committee)	___2___________

For peer review only - http://bmjopen.bmj.com/site/about/guidelines.xhtml

**Introduction**			
Background and rationale	6a	Description of research question and justification for undertaking the trial, including summary of relevant studies (published and unpublished) examining benefits and harms for each intervention	___5__________
	6b	Explanation for choice of comparators	__5___________
Objectives	7	Specific objectives or hypotheses	__6___________
Trial design	8	Description of trial design including type of trial (eg, parallel group, crossover, factorial, single group), allocation ratio, and framework (eg, superiority, equivalence, noninferiority, exploratory)	__6___________
**Methods: Participants, interventions, and outcomes**			
Study setting	9	Description of study settings (eg, community clinic, academic hospital) and list of countries where data will be collected. Reference to where list of study sites can be obtained	__6___________
Eligibility criteria	10	Inclusion and exclusion criteria for participants. If applicable, eligibility criteria for study centres and individuals who will perform the interventions (eg, surgeons, psychotherapists)	_7___________
Interventions	11a	Interventions for each group with sufficient detail to allow replication, including how and when they will be administered	__6 and Figure 1
	11b	Criteria for discontinuing or modifying allocated interventions for a given trial participant (eg, drug dose change in response to harms, participant request, or improving/worsening disease)	__7___________
	11c	Strategies to improve adherence to intervention protocols, and any procedures for monitoring adherence (eg, drug tablet return, laboratory tests)	__8-9_________
	11d	Relevant concomitant care and interventions that are permitted or prohibited during the trial	__9__________
Outcomes	12	Primary, secondary, and other outcomes, including the specific measurement variable (eg, systolic blood pressure), analysis metric (eg, change from baseline, final value, time to event), method of aggregation (eg, median, proportion), and time point for each outcome. Explanation of the clinical relevance of chosen efficacy and harm outcomes is strongly recommended	__11_________
Participant timeline	13	Time schedule of enrolment, interventions (including any run-ins and washouts), assessments, and visits for participants. A schematic diagram is highly recommended (see Figure)	__11 and table 1_

For peer review only - http://bmjopen.bmj.com/site/about/guidelines.xhtml

Sample size	14	Estimated number of participants needed to achieve study objectives and how it was determined, including clinical and statistical assumptions supporting any sample size calculations	___8________
Recruitment	15	Strategies for achieving adequate participant enrolment to reach target sample size	___8-9_________

Methods: Assignment of interventions (for controlled trials)

Allocation:

Sequence generation	16a	Method of generating the allocation sequence (eg, computer-generated random numbers), and list of any factors for stratification. To reduce predictability of a random sequence, details of any planned restriction (eg, blocking) should be provided in a separate document that is unavailable to those who enrol participants or assign interventions	___8_________
Allocation concealment mechanism	16b	Mechanism of implementing the allocation sequence (eg, central telephone; sequentially numbered, opaque, sealed envelopes), describing any steps to conceal the sequence until interventions are assigned	___8_________
Implementation	16c	Who will generate the allocation sequence, who will enrol participants, and who will assign participants to interventions	__8________
Blinding (masking)	17a	Who will be blinded after assignment to interventions (eg, trial participants, care providers, outcome assessors, data analysts), and how	__8_________
	17b	If blinded, circumstances under which unblinding is permissible, and procedure for revealing a participant's allocated intervention during the trial	__8___________

Methods: Data collection, management, and analysis

Data collection methods	18a	Plans for assessment and collection of outcome, baseline, and other trial data, including any related processes to promote data quality (eg, duplicate measurements, training of assessors) and a description of study instruments (eg, questionnaires, laboratory tests) along with their reliability and validity, if known. Reference to where data collection forms can be found, if not in the protocol	__10-11_______
	18b	Plans to promote participant retention and complete follow-up, including list of any outcome data to be collected for participants who discontinue or deviate from intervention protocols	__10-11________

For peer review only - http://bmjopen.bmj.com/site/about/guidelines.xhtml

Data management	19	Plans for data entry, coding, security, and storage, including any related processes to promote data quality (eg, double data entry; range checks for data values). Reference to where details of data management procedures can be found, if not in the protocol	____11________
Statistical methods	20a	Statistical methods for analysing primary and secondary outcomes. Reference to where other details of the statistical analysis plan can be found, if not in the protocol	____12_______
	20b	Methods for any additional analyses (eg, subgroup and adjusted analyses)	____NA_______
	20c	Definition of analysis population relating to protocol non-adherence (eg, as randomised analysis), and any statistical methods to handle missing data (eg, multiple imputation)	___ NA _______
**Methods: Monitoring**			
Data monitoring	21a	Composition of data monitoring committee (DMC); summary of its role and reporting structure; statement of whether it is independent from the sponsor and competing interests; and reference to where further details about its charter can be found, if not in the protocol. Alternatively, an explanation of why a DMC is not needed	___8-9_________
	21b	Description of any interim analyses and stopping guidelines, including who will have access to these interim results and make the final decision to terminate the trial	____8-9______
Harms	22	Plans for collecting, assessing, reporting, and managing solicited and spontaneously reported adverse events and other unintended effects of trial interventions or trial conduct	____8-9______
Auditing	23	Frequency and procedures for auditing trial conduct, if any, and whether the process will be independent from investigators and the sponsor	____8-9_______
**Ethics and dissemination**			
Research ethics approval	24	Plans for seeking research ethics committee/institutional review board (REC/IRB) approval	____13______
Protocol amendments	25	Plans for communicating important protocol modifications (eg, changes to eligibility criteria, outcomes, analyses) to relevant parties (eg, investigators, REC/IRBs, trial participants, trial registries, journals, regulators)	__13_______

For peer review only - http://bmjopen.bmj.com/site/about/guidelines.xhtml

Consent or assent	26a	Who will obtain informed consent or assent from potential trial participants or authorised surrogates, and how (see Item 32)	____13_______
	26b	Additional consent provisions for collection and use of participant data and biological specimens in ancillary studies, if applicable	___13________
Confidentiality	27	How personal information about potential and enrolled participants will be collected, shared, and maintained in order to protect confidentiality before, during, and after the trial	___13________
Declaration of interests	28	Financial and other competing interests for principal investigators for the overall trial and each study site	___2________
Access to data	29	Statement of who will have access to the final trial dataset, and disclosure of contractual agreements that limit such access for investigators	_____________
Ancillary and post-trial care	30	Provisions, if any, for ancillary and post-trial care, and for compensation to those who suffer harm from trial participation	___13________
Dissemination policy	31a	Plans for investigators and sponsor to communicate trial results to participants, healthcare professionals, the public, and other relevant groups (eg, via publication, reporting in results databases, or other data sharing arrangements), including any publication restrictions	___13_________
	31b	Authorship eligibility guidelines and any intended use of professional writers	___13__________
	31c	Plans, if any, for granting public access to the full protocol, participant-level dataset, and statistical code	____13_________
**Appendices**			
Informed consent materials	32	Model consent form and other related documentation given to participants and authorised surrogates	____NA_______
Biological specimens	33	Plans for collection, laboratory evaluation, and storage of biological specimens for genetic or molecular analysis in the current trial and for future use in ancillary studies, if applicable	_____NA____

*It is strongly recommended that this checklist be read in conjunction with the SPIRIT 2013 Explanation & Elaboration for important clarification on the items. Amendments to the protocol should be tracked and dated. The SPIRIT checklist is copyrighted by the SPIRIT Group under the Creative Commons "Attribution-NonCommercial-NoDerivs 3.0 Unported" license.

For peer review only - http://bmjopen.bmj.com/site/about/guidelines.xhtml
